# Consequences of suspected heparin-induced thrombocytopenia in the ICU

**DOI:** 10.1186/cc11030

**Published:** 2012-03-20

**Authors:** M Ostermann, L McIntyre, F Lauzier, J Alhashemi, I Qushmaq, S Langevin, P Dodek, M Albert, K Khwaja, J Kutsiogiannis, L Burry, J Granton, J Friedrich, N Ferguson, J Marshall, S Finfer, D Heels-Ansdell, N Zytaruk, D Cook, J Sheppard, T Warkentin, M Crowther

**Affiliations:** 1 King's College London, UK; 2 University of Ottawa, Canada; 3 CHA-Hôpital de l'Enfant-Jésus, Université Laval, Québec, Canada; 4 King AbdulAziz University, Jeddah, Saudi Arabia; 5 King Faisal Hospital, Jeddah, Saudi Arabia; 6 University of British Columbia, Vancouver, Canada; 7 Université de Montréal, Canada; 8 Université McGill, Montréal, Canada; 9 University of Alberta, Edmonton, Canada; 10 University of Toronto, Canada; 11 The George Institute, Sidney, Australia; 12 McMaster University, Hamilton, Canada

## Introduction

Clinical suspicion of heparin-induced thrombocytopenia (HIT) may prompt changes in drug management and alert clinicians to an increased risk of thrombosis. However, thrombocytopenia in the ICU occurs in about 50% of patients, is multifactorial and is due to HIT in <1%. We aimed to describe the consequences of suspected HIT among medical-surgical critically ill patients in terms of drug and device management, and thrombotic outcomes.

## Methods

We enrolled 3,746 patients in the PROTECT trial comparing prophylactic dalteparin to unfractionated heparin. We defined HIT as occurring in patients with a clinical or laboratory-driven suspicion of HIT and a positive serotonin release assay (SRA). We defined suspected HIT as patients whose clinicians were sufficiently concerned about HIT to withhold heparin. We defined consequences of HIT as occurring from 1 day before it was suspected to 30 days thereafter.

## Results

One hundred and thirty patients (3.5%) had heparin held due to clinical suspicion of HIT. Of these, 10 (7.7%) had a positive SRA test. The drugs and devices used for thromboprophylaxis, as well as thrombotic events, are outlined in Table 1. At least one new thrombotic event developed in 23.8% of patients with suspected HIT and 40.0% of patients with HIT.

**Table 1:** 

	1 day before to 30 days after heparin held for suspect HIT
Intervention	
Danaparoid	34 (26.2)
Lepirudin	8 (6.2)
Fondaparinux	11 (8.5)
Argatroban	19 (14.6)
Any of the above drugs	67 (51.5)
Anti-embolic stockings	25 (19.2)
Pneumatic compression device	37 (28.5)
Anti-embolic stockings or pneumatic compression device	49 (37.7)
Any of the above interventions	96 (73.8)
Incident thromboses	
Venous thrombosis (including PE)	30 (23.1)
Arterial thrombosis	1 (0.8)
Progression of a previous thrombus	2 (1.5)
Any of the above	31 (23.8)

## Conclusion

Over 90% of patients with suspected HIT did not have HIT. One-half of patients with suspected HIT were prescribed another anticoagulant and one-third received mechanical prophylaxis. Thrombotic rates are higher in patients with HIT and suspected HIT than other patients. The frequent suspicion of HIT in critically ill patients and initiation of other interventions may create a greater clinical and economic burden than HIT itself.

